# Design and Synthesis of Some Novel 4-(4-substituted aryl) Semicarbazones as Anticonvulsant Agents

**DOI:** 10.4103/0250-474X.70485

**Published:** 2010

**Authors:** Anita Singh, C. Pande, P. Gahtori, S. N. Pandeya, J. P. Stables

**Affiliations:** Department of Pharmacy, Kumaun University, Bhimtal-263 002, India; 1 Saroj institute of Pharmacy, Lucknow-222 001, India; 2 Epilepsy Branch, National Institute of Neurological Disorders and Stroke, National Institutes of Health, Bethesda, MD 20892, USA

**Keywords:** Anticonvulsant, hydrogen bonding domain, lipophilic aryl/alkyl ring, 4-substituted semicarbazone

## Abstract

In the present study, a series of 4-(4-substituted aryl) semicarbazones were synthesized from substituted anilines and subsequently evaluated for their anticonvulsant activities. The anticonvulsant activities were established by the anticonvulsant drug development (ADD) programme NIH, USA using experimental animal, adult male FCM mice (20–25 g) and adult Sprague-Dawley rats (100–150 g) and screened against electroshock seizure, subcutaneous metrazole and minimal neurotoxicity tests in mice. Compound 7 was found equipotent to carbamazepine in both MES and ScPTZ tests. This study has highlighted the importance of distal alkyl chain which influences the anticonvulsant activity.

Epilepsy is the most common serious neurological disorder worldwide[1]. The anticonvulsant properties have been displayed by various hydrazones[2], amides[3] and carbamides[4]. The prime need was to search a molecule which could complement to all these structures. Recently, Unverferth *et al*. suggested a pharmacophore model for structurally different anticonvulsant containing aryl ring, electron donor and hydrogen bonding functions[5].

The lipophilic aryl ring with chloro, bromo or nitro groups have been found to be essential for anticonvulsant activity. The distal aryl ring is also implicated at the binding site. The hydrogen bonding in semicarbazone series has been suggested by Dimmock *et al*.[6,7] to be the terminal -NHCONH_2_ group. In continuation, Pandeya *et al*.[8,9] suggested HBD to be adjacent to the lipophilic aryl ring and another hydrophobic centre for activity. Generally, the structural requirements for activity in the maximal electroshock (MES) screen have been found the presence of a large hydrophobic group (phenytoin) and for subcutaneous pentylene tetrazole (ScPTZ) screen, a smaller and less hydrophobic group (ethosuximide) near to two electron-donor atoms[10]. In this report, here we demonstrate the lipophilic aryl ring substitution in semicarbazones along with nitro, chloro and bromo groups as novel anticonvulsant agents.

Melting points of the synthesized compounds were taken by the open capillary method and are uncorrected. UV (EtOH-Band λ_max_, nm) and IR (KBr, cm^−1^) spectra were consistent with the assigned structures recorded on Jasco Model 7800 and Jasco FT/IR-5300 instruments respectively and ^1^H NMR (DMSO d_6_-TMS, ppm) spectra were recorded Jeol FX 90Q FT spectroscope using TMS as internal standard. Chemical shift are quoted in δ (ppm) units relative to TMS. Purity of the synthesized compounds were checked by TLC using silica gel G (Merck, Germany) and chloroform methanol (9:1) solvent system. 4-substituted semicarbazone derivatives were prepared in three steps as follows:

4-substituted aniline (0.1 mol) was dissolved in 10 ml of glacial acetic acid (GAA) and diluted to 100 ml with water. Equimolar quantity (0.1 mol) of sodium cyanate in 50 ml of warm water was added in previous solution with stirring. The reaction mixture was allowed to stand for 30 min, and then it was filtered, washed, dried and recrystallized from boiling water.

To a solution of 4-substituted phenyl urea (0.1 mol) in 20 ml ethanol, an equimolar (0.1 mol) quantity of hydrazine hydrate was added. The reaction mixture was made alkaline by adding 4 g of NaOH, was refluxed for 1-2 h and cooled in ice. The product was filtered under suction and recrystallised from ethanol.

A solution of p-phenyl semicarbazide (0.01 mol) and an equimolar (0.01 mol) quantity of appropriate carbonyl compound was refluxed for 30 min in the presence of glacial acetic acid (1-1.5 ml). The product obtained after cooling was filtered and recrystallised from 95% ethanol to give pure compounds (Scheme 1). The characterization data of the compounds are reported (Table 1).

**Scheme 1:**
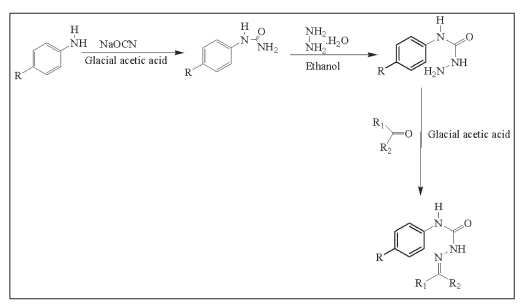
Synthesis of 4-(4-substituted aryl) semicarbazones (1-10) R=Cl, F, NO^2^, Br and R^1^,R^2^= alkyl/ aryl groups

**TABLE 1:** PHYSICAL AND ANALYTICAL CHARACTERISTICS OF 4-(4-SUBSTITUTED ARYL) SEMICARBAZONES (1-10)
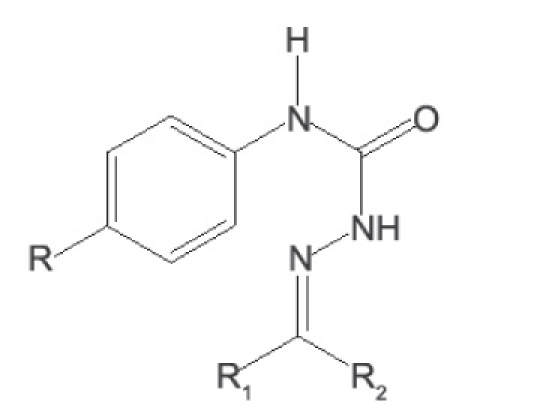

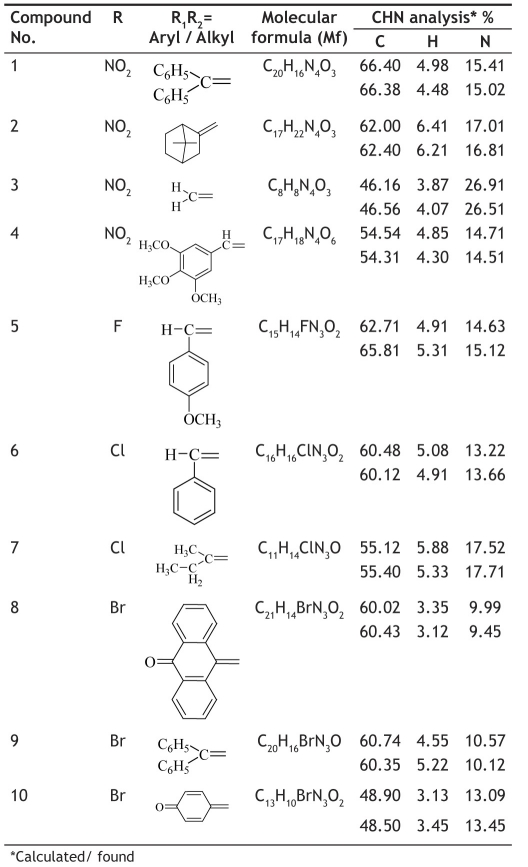

1-(diPhenylmethylene)-4-(4-nitrophenyl)semicarbazone (1); m.p. 123-125°; Yield (%) 70; R_f_ 0.56; UV (EtOH, Band λ_max_ nm) 230, 337, 376; IR (KBr, cm^−1^) 3350 (Ar-NH), 1700(C=O), 1610 (C=N), 820 (Ar-H); ^1^H NMR (DMSO d_6_-TMS, ppm) 5.8 (s, 1H, Ar-NH), 6.3-6.5 (m, 10H, benzophenone ring), 7.6-7.9 (m, 4H, nitrophenyl ring), 8.0 (s, 1H, CONH).

1-(7,7-diMethylbicyclo[2.2.1]heptan-2-ylidene)-4-(4-nitrophenyl)semicarbazone (2); m.p. 145-147°; Yield (%) 65; R_f_ 0.63; UV (EtOH, Band λ_max_nm) 231, 373, 379; IR (KBr, cm^−1^) 3365 (Ar-NH), 1635 (C=O), 1475 (Ar-NO_2_), 845 (Ar-H); ^1^H NMR (DMSO d_6_-TMS, ppm) 5.9 (s, 1H, Ar-NH), 3.4-3.7 (m, 16H, camphor ring), 6.4-6.7 (m, 4H, nitrophenyl ring), 7.6-8.0(d, 2H,NH).

1-Methylene-4-(4-nitrophenyl) semicarbazone (3); m.p. 230-235°; Yield (%) 60; R_f_ 0.59; UV (EtOH, Band λ_max_ nm) 283, 372, 380; IR (KBr, cm^−1^) 3610 (Ar-NH), 1638(C=O), 1470(Ar-NO_2_), 850(Ar-H); ^1^H NMR (DMSO d_6_-TMS, ppm) 5.7 (s, 1H, Ar-NH), 6.9-7.1 (m, 4H, nitrophenyl ring), 2.8-2.9 (d, 2H, H_2_C= group), 8.1 (s, 1H, CONH).

1-(3,4,5-triMethoxybenzylidene)-4-(4-nitrophenyl)semicarbazone (4); m.p. 125-130°; Yield (%) 92; R_f_ 0.48; UV (EtOH, Band λ_max_ nm) ;225, 376; IR (KBr, cm^−1^) 3370 (CONH), 3270 (Ar-NH), 1720 (C=O), 1610 (C=N), 1525 (Ar-NO_2_), 855(Ar-H); ^1^H NMR (DMSO d_6_-TMS, ppm) 6.0 (s, 1H, Ar-NH), 4.01 (s, 3H, 3- methoxy), 3.76 (s, 3H, 4- methoxy), 3.75 (s, 3H, 5- methoxy), 8.1 (s, 1H, CONH).

1-(4-Methoxybenzylidene)-4-(4-fluorophenyl) semicarbazone (5); m.p. 178-180°; Yield (%) 74; R_f_ 0.76; UV (EtOH, Band λ_max_ nm) 280, 236; IR (KBr, cm^−1^) 3430(CONH), 3320(Ar-NH), 1735(C=O), 1570(C=N), 1245(Ar-F), 830 (Ar-H); ^1^H NMR (DMSO d_6_-TMS, ppm) 3.48 (s, 1H), 3.95 (s, 3H, -OCH_3_), 6.0 (s, 1H, Ar-NH), 7.23-7.33 (m, 4H, p-methoxylphenyl), 7.25-7.59 (m, 4H, p-flurophenyl), 10.09 (s, 1H, CONH).

1-Benzylidene-4-(4-chlorophenyl) semicarbazone (6); m.p. 160-163°; Yield (%) 55; R_f_ 0.74; UV (EtOH, Band λ_max_ nm) 328, 228; IR (KBr, cm^−1^) 3370 (sec. NH), 2815 (CH_3_-N), 1682 (C=O), 1560 (C=N), 1312 (C-N amine), 1035 (Ar-Cl), 845 (Ar-H); ^1^H NMR (DMSO d_6_-TMS, ppm) 3.42 (s, 3H, N-CH_3_), 3.92 (s, 3H, OCH_3_phenyl), 7.85-8.05 (m, 4H, p-chlorophenyl), 8.83 (s, 1H, Ar-NH), 7.23-7.26 (m, 4H, p-nitrophenyl), 8.06 (s, 1H, CONH).

1-(Butan-2-ylidene)-4-(4-chlorophenyl) semicarbazone (7); m.p. 154-157°; Yield (%) 65; R_f_ 0.77; UV (EtOH, Band λ_max_ nm) 228, 251, 374; IR (KBr, cm^−1^) 3420 (sec. NH, Ar-NH), 3320 sec. NH, amide), 1735 (C=O), 1570 (C=N), 1245 (Ar-Cl), 830 (Ar-H); ^1^H NMR (DMSO d_6_-TMS, ppm) 2.29 (s, 7H, ethylmethyl ketone), 5.8 (s, 1H, Ar-NH), 7.2-7.3 (m, 4H, 4-chlorophenyl), 8.8 (s, 1H, CONH).

4-(4-Bromophenyl)-1-(9-oxoanthracen-10(9H)-ylidene) semicarbazone (8); m.p. 207-211°; Yield (%) 30; R_f_ 0.49; UV (EtOH, Band λ_max_ nm) 251, 283, 372, 380; IR (KBr, cm^−1^); 3372 (Ar-NH), 1630(C=O), 1575(C=N), 1472 CH_2_ def.), (840 (Ar-H); ^1^H NMR (DMSO d_6_-TMS, ppm) 6.1 (s, 1H, Ar-NH), 7.35-7.9 (m, 8H, anthraquinone ring), 7.12-7.56 (m, 4H, p-bromophenyl), 8.88 (s, 1H, CONH).

4-(4-Bromophenyl)-1-(diphenylmethylene) semicarbazone (9); m.p. 150-156°; Yield (%) 37; R_f_ 0.52; UV (EtOH, Band λ_max_ nm) 251, 271, 324; IR (KBr, cm^−1^) 3365(Ar-NH), 1572 (C=N), 1478 (CH_2_ def.), 800 (Ar-H); ^1^H NMR (DMSO d_6_-TMS, ppm) 5.9 (s, H), 6.4-6.6 (m, 10 H, benzophenone ring), 8.80 (s, 1H, CONH).

4-(4-Bromophenyl)-1-(4-oxocyclohexa-2,5-dienylidene)semicarbazones (10); m.p. 147-150°; Yield (%) 35; R_f_0.53; UV (EtOH, Band λ_max_ nm) 260, 266, 272; IR (KBr, cm^−1^) 3372 (Ar-NH), 1635 (C=O), 1572(C=N), 820(Ar-H); ^1^H NMR (DMSO d_6_-TMS, ppm) 6.0 (s, 1H, Ar-NH), 6.5-6.9 (m, 4H, quinone ring), 8.1 (m, 4H-bromophenyl ring), 9.8 (s, 1H, CONH).

The anticonvulsant activities were established by the anticonvulsant drug development (ADD) programme[11]. Adult male FCM mice (20–25 g) and adult male Sprague Dawley rats (100–150 g) were used as experimental animals. The animals were maintained on an adequate diet and allowed free access to food and water except during the short time they are removed from their cages for testing. The animals were maintained at room temperature (25–30°). Number of animals was used from 4-6 depending upon the nature of test.

Initially all the compounds dissolved in polyethylene glycol were administered intraperitoneally (0.01 ml /g body weight in mice and 0.004 ml /g body weight in rats) at a dose of 30, 100, and 300 mg/kg to all the animals respectively. Compounds were screened for anticonvulsant activity by maximal electroshock seizure (MES), subcutaneous metrazole (ScMet) and Minimal neurotoxicity (TOX) test in mice.

MES or maximal seizure pattern test, in which an electrical stimulus of 0.2 s in duration (50 mA in mice and 150 mA in rat at 60Hz) was delivered via corneal electrodes primed with an electrolyte solution containing an anesthetic agent. Mice are tested at 30 min and 4 h following doses of 30, 100 and 300 mg/kg of test compound. Abolition of the hindlimb tonic extensor component indicates the test compound’s ability to inhibit MES-induced seizure spread[12].

The subcutaneous pentylenetetrazol (metrazol) seizure test (scPTZ) was performed using a dose of pentylenetetrazol (70 mg/kg in Sprague Dawley rats) to produce clonic seizures lasting for a period of at least five seconds in 97 per cent (CD97) of animals tested. At the anticipated time of testing the convulsant is administered subcutaneously. The test compound is administered intraperitoneally in mice and orally in rats. Animals are observed over a 30 min period. Absence of clonic spasms in the observed time period indicates a compound’s ability to abolish the effect of pentylenetetrazol on seizure threshold[13].

Minimal neurotoxicity (TOX) was evaluated in rats by examining them for behavioral toxicity using the positional sense test and a gait and stance test. The mice were trained to stand on an accelerating rotarod of diameter 3.2 cm, which rotated at 10 rev/min. Animals were given intraperitoneal injections of the test compounds in the doses of 30, 100 and 300 mg/ kg body weight. Neurotoxicity was indicated by the inability of the animal to maintain equilibrium on the rod for at least one minute in each of the three trials[14].

The UV spectrum shows presence of chromophores in the 4-substituted aryl/alkyl semicarbazones. The benzene ring absorption is in the range of 200-205 nm pertaining to K-band. The π- π^*^transition. K-band of substituted p-nitroaniline was very strong λ_max_ 375–380 nm. The halo substituted derivatives absorbed at 265 nm. The formation of the substituted hydrazones and semicarbazones were confirmed with the formation of C=N band in all spectra from 1500–1620 cm^−1^. The CONH stretching for amides was observed in the 4-substituted aryl/alkyl semicarbazones at about 3180-3320 cm^−1^. The chemical shift for aromatic amino group (Ar-NH) was found at 5.82 to 5.89 ppm and 8.86-8.91 ppm for -CONH group present in semicarbazone. The signals for aromatic protons were observed at 7.2-7.6 ppm.

The series of 4-subsituted phenyl semicarbazones were found to possess significant anticonvulsant activities (Table 2). The compounds were found to be active at different doses in the MES test excluding compound 3 and 6. Compounds 4, 7 and 8 were active in MES test (100 mg/kg), remaining other compounds 1, 2, 5, 9 and 10 showed anticonvulsant activity in MES test at (300 mg/kg). Compounds 4, 5, 7, 9 and 10 exhibited activity in ScPTZ test at a dose of (100 mg/kg). Compounds 4 and 7 were effective in both MES and ScPTZ tests. Compound 7 was further evaluated in MES test upon oral administration (30 mg/kg) in rats which gave protection 75% up to an extended period of 4 hours. Compound 7 was reported no neurotoxicity at a dose of 30 mg/kg. Absence of hydrophobic side at the distal ring (Compound 3), did not exhibit any type of anticonvulsant activity. Replacement of both hydrogens in 3, by alkyl/aryl groups produced compounds active in both MES and ScPTZ tests and further replacement of one of the hydrogen with an aryl ring in compound 5 produce activity in ScPTZ tests. Compound 7 was also examined for oral activity in the MES screening (Table 3). The figures reveal the lowest dose at which bioactivity was demonstrated and the lines indicate the absence of anticonvulsant activity.

**TABLE 2:** ANTICONVULSANT ACTIVITY OF 4-(4-SUBSTITUTED ARYL) SEMICARBAZONES IN MICE

Compound No.	Concentration (mg/ kg body weight)				
	MES^a^		ScMet^b^	TOX^c^	
	0.5 h	4 h	0.5 h	0.5 h	4 h
1	300	--	--	30 (50 % d) 100 (65%) 300 (100%)	100 (50%) 100 (33%)-5h
2	300 (100%)	--	--	--	--
3	--	--	--	--	100
4	100 (33%)	--	100	100	--
5	--	--	100	--	--
6	--	--	--	--	--
7	100 (60%) 300 (100%)	300 (100%)	100 (60%) 300 (100%)	30 (50%) 100 (75%) 300 (100%)	100 (25%) 300 (50%)
8	100 (100%) 300 (100%)	300 (100%)	300 (66%)	100 (75%) 300 (100%)	100 (100%) 300 (100%)
9	300 (25%)	--	100 (25%)	300 (75%)	300 (100%)
10	300 (75%)	100 (25%)	--	100 (50%)	300 (75%)
Phenytoin	30	30	--	100	100
Carbamazepine	30	100	100	100	300

a Maximal electroshock seizure

b Subcutaneous metrazole

c Neurotoxicity

d Percent protection

Doses tested were 30, 100 AND 300 mg/ kg

**TABLE 3:** MES TEST UPON ORAL ADMINISTRATION OF COMPOUND 7 IN RATS

Time (h)	0.25	0.5	1.0	2.0	4.0
MESα	2/4	1/4	3/4	3/4	3/4
TOXβ	0/4	0/4	0/4	0/4	0/4

a Number of animals affected/ number of animals used

α Maximal electroshock seizure

β Neurotoxicity, Compound 7 was administered at a dose of 30 mg kg^-1^ in rats^a^.

Generally MES active compounds are considered to exhibit activity in grandmal epilepsy, contain larger hydrophobic center (phenytoin)[15] and ScMet active compounds are considered petitmal active, contain smaller hydrophobic centers (ethosuximide)[16]. This study has highlighted the importance of distal alkyl chain which influences the anticonvulsant activity. The distal hydrophobic side perhaps may be differentiating the kind of anticonvulsant activity. Compound 7 was found equipotent to carbamazepine in both MES and ScPTZ tests. In oral administration test it has not shown any neurotoxicity. It may be considered as the lead molecule for further optimization work in the anticonvulsant activity.
